# Inhibition of Caspase 1 Reduces Blood Pressure, Cytotoxic NK Cells, and Inflammatory T-Helper 17 Cells in Placental Ischemic Rats

**DOI:** 10.3390/ijms25020863

**Published:** 2024-01-10

**Authors:** Corbin A. Shields, Geilda A. Tardo, Xi Wang, Gregory Peacock, Marcus Robbins, Hannah Glenn, Rachel Wilson, Jan M. Williams, Denise C. Cornelius

**Affiliations:** 1 Department of Pharmacolocy and Toxicology, University of Mississippi Medical Center, Jackson, MS 39216, USA; cashields@umc.edu (C.A.S.); geildaann@gmail.com (G.A.T.); xwang3@umc.edu (X.W.); mkrobbins@umc.edu (M.R.); hlglenn@umc.edu (H.G.); rwilson15@umc.edu (R.W.); jmwilliams5@umc.edu (J.M.W.); 2 Department of Emergency Medicine, University of Mississippi Medical Center, Jackson, MS 39216, USA; gregp1906@gmail.com

**Keywords:** preeclampsia, inflammation, Caspase 1

## Abstract

Preeclampsia (PE) is characterized by maternal hypertension, fetal growth restriction (FGR), and increased inflammation and populations of cytotoxic NK cells (cNKs) and inflammatory T-Helper 17 cells (TH17s). Both cytotoxic NK cells and TH17 cells are heavily influenced via IL-1β signaling. Caspase 1 activity leads to the release of the inflammatory cytokine IL-1β, which is increased in women with PE. Therefore, we tested the hypothesis that the inhibition of Caspase 1 with VX-765 in rats with reduced uterine perfusion pressure (RUPP) will attenuate PE pathophysiology. On gestation day (GD) 14, timed pregnant Sprague–Dawley rats underwent the RUPP or Sham procedure and were separated into groups that received either vehicle or VX-765 (50 mg/kg/day i.p.). On GD19, MAP was measured via carotid catheter and blood and tissues were collected. Bio-Plex and flow cytometry analysis were performed on placental tissues. Placental IL-1β was increased in the RUPP rats vs. the Sham rats and treatment with VX-765 reduced IL-1β in the RUPP rats. Caspase 1 inhibition reduced placental cNKs and TH17s in RUPP rats compared to vehicle-treated RUPP rats. Increased MAP was observed in RUPP rats compared with Sham rats and was reduced in RUPP + VX-765 rats. Placental reactive oxygen species (ROS) were elevated in RUPP rats compared to Sham rats. VX-765 administration reduced ROS in treated RUPP rats. Caspase 1 inhibition increased the number of live pups, yet had no effect on fetal weight or placental efficiency in the treated groups. In conclusion, Caspase 1 inhibition reduces placental IL-1β, inflammatory TH17 and cNK populations, and reduces MAP in RUPP rats. These data suggest that Caspase 1 is a key contributor to PE pathophysiology. This warrants further investigation of Caspase 1 as a potential therapeutic target to improve maternal outcomes in PE.

## 1. Introduction

Preeclampsia (PE) is defined as a maternal blood pressure ≥ 140/90 mmHg after 20 weeks of gestation in conjunction with one or more coexisting complications including proteinuria, maternal organ dysfunction, abnormal uterine artery Doppler sonography, and potential fetal growth restriction [1]. For years, preeclampsia (PE) has served as a major health risk affecting almost 3–8% of pregnancies in the United States [2]. In our own state of Mississippi, Hypertensive Disorders of Pregnancy (HDP), which include PE, account for 6.5% of pregnancy-related deaths, which is more than double the national rate (2.5%) [3]. Despite major efforts, there is currently still no cure beyond delivery of the child and the placenta. The effects of pregnancies complicated by PE do not stop there, however, as PE serves as a major risk factor for the development of cardiovascular disease (CVD) for both the mother and child later in life [4]. Work from our lab and others has aimed to characterize the mechanisms of pathophysiology in PE and identify potential biomarkers and therapeutic targets to aid in the diagnosis and treatment of PE.

The maternal immune system strikes a critical balance during gestation between protection from pathogens and inducing tolerance to the developing fetus and placenta. PE patients, however, display a chronic state of immune activation that increases the expression of inflammatory cytokines, antiangiogenic factors, and increased oxidative stress [5,6]. Increased populations of effector cells, including proinflammatory T-helper 17 cells (TH17s) and cytolytic NK cells (cNKs), and decreased populations of immune-tolerant cells are seen in PE patients, marking a shift from the tolerant immune profile [7,8]. Our previous work demonstrates a role for these immune cells in mediating the pathophysiology of PE, including fetal growth restriction, hypertension, inflammation, and placental oxidative stress [9,10,11,12]. The placental NOD-like receptor and pyrin domain-containing protein 3 (NLRP3) inflammasome is a multiprotein complex composed of NLRP3, apoptosis-associated speck-like protein containing a caspase recruitment domain (ASC), and Caspase 1 as an IL-1β-converting enzyme [13]. NLRP3 activity initiates pyroptosis, triggers proinflammatory cytokine release, and has been shown to be increased in the placentas of women with PE [14,15]. In other diseases, the NLRP3 inflammasome has been shown to increase effector immune cells like TH17s and cNKs [16,17,18], both of which are elevated in PE [7]. Our recent study found that inhibiting the activation of NLRP3 had beneficial effects in a reduced uterine perfusion pressure (RUPP) rat model of placental ischemia [19]. The RUPP rat is a commonly used and well-characterized preclinical model of placental ischemia that recapitulates many of the characteristics of PE, including maternal hypertension, fetal growth restriction, and chronic inflammation [20].

In the present study, we hypothesize that inhibiting the enzymatic activity of the NLRP3 inflammasome by blocking Caspase 1 will reduce the effector T and NK cells, thereby reducing inflammation and oxidative stress, yielding a concurrent improvement in maternal and fetal outcomes. Using the RUPP rat model of placental ischemia, we inhibited Caspase 1 with Belnacasan (VX-765) and assessed its effect on TH17 and cNK activation, IL-1β, and PE pathophysiology.

## 2. Results

### 2.1. Caspase 1 Inhibition Reduces Circulating IL-1β in RUPP Rats

**Figure 1:**
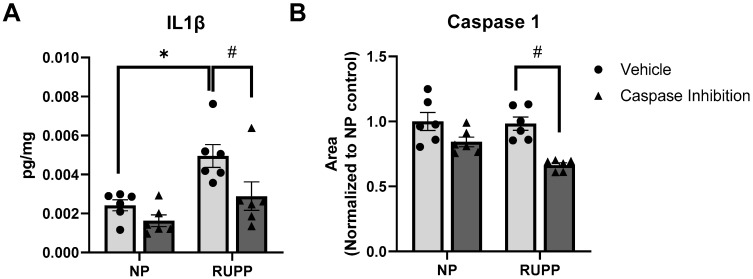
VX-765 decreased placental IL-1β and placental Caspase 1 expression in RUPP rats. Placental IL-1β (**A**) (N = 6) was assessed via Bio-Plex. Caspase 1 (**B**) (N = 6) expression was measured via Western blot. Data are presented as mean ± SEM. * *p* < 0.05 vs. vehicle NP rats, # *p* < 0.05 vs. vehicle RUPP rats. NP, normal pregnant; RUPP, reduced uterine perfusion pressure.

Using Bio-Rad’s Bio-Plex singleplex assay, we assessed placental IL-1β levels (Figure 1) as IL-1β is directly downstream of Caspase 1 activation. The RUPP rat’s IL-1β was markedly elevated compared to normal pregnant (NP) rats (4.9 pg/µg vs. 2.5 pg/µg, respectively, *p* = 0.011). The administration of VX-765 significantly reduced IL-1β in RUPP rats to 2.8 pg/µg (*p* = 0.039 vs. vehicle RUPP rats) (Figure 1A), with no changes present in the NP rats. The total Caspase 1 expression was assessed via Western blot. We found no differences in Caspase 1 protein expression between NP and RUPP rats, while RUPP rats treated with VX-765 had decreased expression of Caspase 1 (Figure 1B). These findings suggest that the administered dose was effective at inhibiting Caspase 1 activity.

### 2.2. Maternal Outcomes Improve with Caspase 1 Inhibition

**Figure 2:**
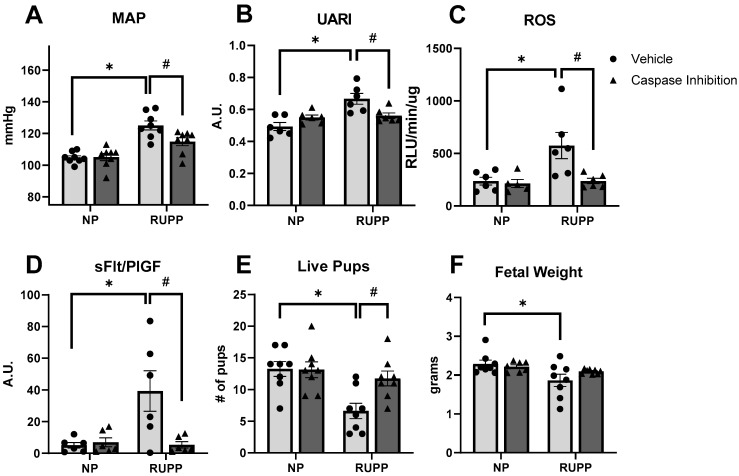
Caspase 1 inhibition reduced maternal MAP, UARI, and ROS and improved fetal survival. MAP (**A**) (N = 8) was measured via carotid catheter, UARI (**B**) (N = 6) via Doppler ultrasound, ROS (**C**) (N = 6) via luciferase assay, sFlt/PlGF (**D**) (N = 6) ratio via ELISA. Number of live pups (**E**) (N = 8) and fetal weight (**F**) (N = 8) were measured at harvest. Data are presented as mean ± SEM. * *p* < 0.05 vs. vehicle NP rats, # *p* < 0.05 vs. vehicle RUPP rats. NP, normal pregnant; RUPP, reduced uterine perfusion pressure.

The primary target of PE therapy is to reduce maternal blood pressure, to prolong the pregnancy and allow the fetus more time to develop. The RUPP model of placental ischemia routinely produces elevated MAP versus NP controls. In this study, the MAP was 125.1 mmhg in the RUPP rats and 104.8 mmhg in the NP controls (*p* < 0.0001) (Figure 2A). Accompanying the elevated MAP, the uterine artery resistance index (UARI) was markedly elevated in the RUPP rats vs. the NP controls (0.667 AU vs. 0.494 0.667 AU vs. 0.494 AU, respectively, *p* = 0.0003) (Figure 2B). The treatment with VX-765 produced a significant reduction in MAP in the RUPP rats (114.9 mmhg, *p* = 0.0143 vs. vehicle RUPP rats), as well as an improvement in uterine artery function by reducing the UARI (0.561 AU, *p* = 0.026 vs. vehicle RUPP rats). No differences in the MAP or UARI were present in the treated NP group. The placental ROS was markedly elevated in the RUPP rats (575 RLU/min/µg vs. 236 RLU/min/µg in NP controls, *p* = 0.029) (Figure 2C). Caspase 1 inhibition reduced placental ROS in the RUPP rats by a significant margin (237.4 RLU/min/µg, *p* = 0.0291 vs. vehicle RUPP rats). VX-765 caused no changes between the NP groups. While no differences were detected in sFlt or PlGF across all groups (Supplemental Figure S1), the RUPP rats displayed an increased sFlt/PlGF ratio compared to NP controls (39.29 A.U. vs. 5.037 A.U., respectively, *p* < 0.01), similar to what is seen clinically in PE patients (Figure 2D). VX-765 returned the sFlt/PlGF levels in treated RUPP rats to near the NP rat’s levels (5.338 A.U., *p* < 0.01 vs. vehicle RUPP rats). The total litter size (number of live pups) was reduced in the RUPP rats compared to the NP controls (13.25 pups vs. 6.625 pups, *p* < 0.01) (Figure 2E). Caspase 1 inhibition increased pup survival in the VX-765-treated RUPP rats (11.75 pups, *p* = 0.02 vs. vehicle RUPP rats). The fetal weight was reduced in the RUPP rats, as expected (1.86 g vs. 2.28 g in NP controls, *p* = 0.029) (Figure 2F); however, treatment with VX-765 did not increase the fetal weight in the RUPP rats, despite the improved MAP and UARI. Furthermore, no differences in placental weight were observed among any of the groups (Supplemental Figure S2).

### 2.3. Caspase 1 Inhibition Reduces Circulating and Tissue-Resident Populations of TH17 Cells and Cytotoxic NK Cells

**Figure 3:**
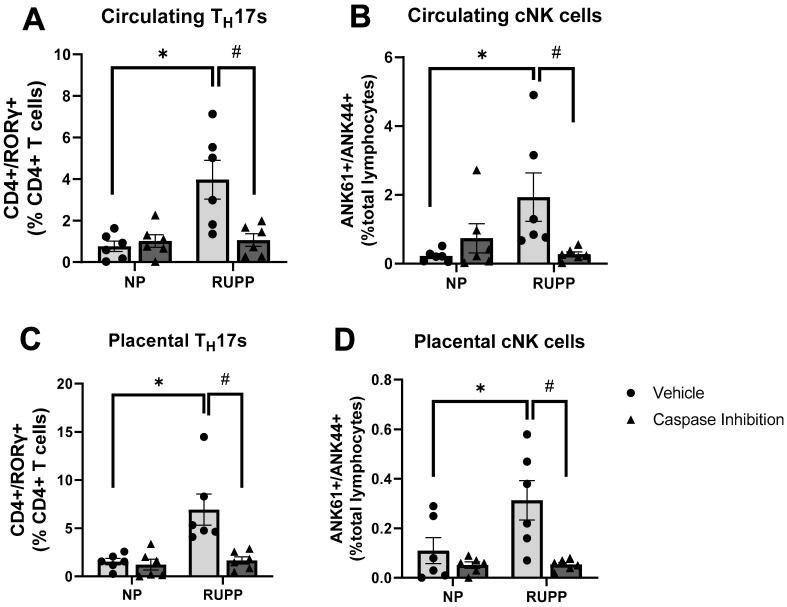
Inhibition of Caspase 1 resulted in a reduction in both circulating and tissue-resident TR_H_R17 and cNK cells in RUPP rats. Circulating TR_H_R17s (**A**) (N = 6), circulating cNKs (**B**) (N = 6), placental TR_H_R17s (**C**) (N = 6), and placental cNKs (**D**) (N = 6) were all assessed via flow cytometry. Data are presented as mean ± SEM. * *p* < 0.05 vs. vehicle NP rats, # *p* < 0.05 vs. vehicle RUPP rats. NP, normal pregnant; RUPP, reduced uterine perfusion pressure.

In concurrence with our previous report [19], this cohort of RUPP rats displayed elevated populations of activated cNK and TH17 cells compared to their NP counterparts (Figure 3). Circulating cNK cells comprised 1.935% of the total lymphocytes in the RUPP rats vs. 0.22% in the NP controls (*p* = 0.048) (Figure 3B). Circulating cNK cells were markedly reduced with VX-765 administration in the RUPP rats (0.27%total lymphocytes, *p* = 0.048 vs. vehicle RUPP rats). Placental cNK cells followed the same pattern. The placentas from RUPP rats showed higher cNK cells (0.362%total lymphocytes) than those from NP controls (0.116%total lymphocytes, *p* = 0.007) (Figure 3D). Caspase 1 inhibition significantly reduced the placental cNK cells in the RUPP rats (0.053%total lymphocytes, *p* = 0.0009 vs. vehicle RUPP rats). No changes in circulating or tissue cNK cells were present in the NP groups, regardless of treatment. The TH17 cells were significantly elevated in the circulation of the RUPP rats compared to the NP controls (3.972%CD4+ T cells vs. 0.761%CD4+ T cells, *p* = 0.002) (Figure 3A). VX-765 reduced circulating TH17 cells in the RUPP rats to 1.065%CD4+ T cells (*p* = 0.003 vs. vehicle RUPP rats). Placental TH17 cells were higher in the RUPP rats (7.25%CD4+ T cells) than in their NP counterparts (1.528%CD4+ T cells, *p* = 0.003) (Figure 3C). This cell population was also reduced by the administration of VX-765 in the RUPP rats (1.663%CD4+ T cells, *p* = 0.003 vs. vehicle RUPP rats). These findings strengthen the claim that NLRP3 activation is related to effector immune cell expression in PE.

## 3. Discussion

NLRP3 expression is reported to have a role in mediating inflammation and vascular function in PE as well as other hypertensive disorders [21,22,23,24,25]. Previous work from our lab demonstrated that NLRP3 expression is elevated in the RUPP rat model of placental ischemia; we uncovered a cause-and-effect relationship between NLRP3 inflammasome activation and effector cell populations by inhibiting NLRP3 activation with MCC950 and esomeprazole [19]. The current study aimed to build on those findings by inhibiting the activity of the enzyme Caspase 1, a critical member of the NLRP3 inflammasome. The administration of VX-765 significantly reduced the protein expression of Caspase 1 only in treated RUPP rats. VX-765 also reduced the placental levels of IL-1β, a direct downstream cytokine of Caspase 1 activity, in RUPP rats, similar to the levels in NP controls. There were no changes in the IL-1β levels in NP rats, possibly due to the fact that Caspase 1 activity is low in the NP condition (based on the low IL-1β levels), and the administered dose had little effect. We found that administration of the Caspase 1 inhibitor, VX-765, significantly reduces circulating and tissue populations of effector TH17 cells and cNK cells. This is accompanied by a marked reduction in maternal MAP, UARI, and placental ROS, alongside an improved sFlt/PlGF ratio and live litter size. Previous reports showed that RUPP rats display elevations in sFlt and reduced PlGF [20]. In the current cohort of animals, sFlt was 23% higher in the RUPP rats than in the NP rats; however, this did not reach statistical significance. Likewise, PlGF in these RUPP animals was decreased 6-fold compared to the NP controls, again not to significance. The high variability of commercially available kits for both sFlt and PlGF could be a possible cause of the lack of significant findings that our limited number of animals were unable to overcome. Importantly, the ratio of soluble fms-like tyrosine kinase 1 (sFlt) to placental growth factor (PlGF) was recently approved by the FDA for clinical utilization as a predictor of preeclampsia risk over either biomarker individually [26]. Our data demonstrate a sFlt/PlGF ratio pattern in RUPP vs. NP rats similar to what is observed in PE vs. normal pregnant patients. Caspase 1 inhibition normalized the sFlt/PlGF ratio in the treated RUPP rats. Taken together, these data determine a direct role of Caspase 1 activity in regulating immune cells and angiogenic factors in response to placental ischemia. Thus, the role of Caspase 1 and its potential as a therapeutic target to inhibit the pathophysiology of PE warrant further investigation.

Caspases are an evolutionarily conserved family of cysteine-dependent proteases. Caspase activity is involved in many cellular processes, including apoptosis, proliferation, differentiation, and inflammatory response; however, due to their poor efficacy and toxic effects, synthetic caspase inhibitors have had difficulty advancing through clinical trials [27]. Nevertheless, the intricate relationship between caspases and cardiovascular diseases maintains interest in caspases as targets. In mice, Caspase 1 deficiency correlates with a reduction in atherosclerosis, as well as reducing endothelial cell activation and monocyte recruitment to the plaque [28,29]. Inhibition of Caspase 1 has also been shown to reduce myocardial infarction size in a rat ischemia–reperfusion injury model [30]. Important to our field, Caspase 1 activity is implicated in the mechanisms leading to spontaneous term parturition and preterm labor [31,32,33].

Both human and murine Caspase 1 are involved in the processing and release of the proinflammatory cytokine IL-1β [34,35]. Caspase 1 exists in the cytosol as a zymogen [36,37]. Once activated, the dormant procaspase-1 zymogen self-cleaves into the enzymatically active heterodimer [38]. Active Caspase 1 is necessary for the cleavage of pro-IL-1β and pro-IL-18 into their mature, active forms. Caspase 1, IL-1β, and IL-18 are all increased in the placentas of women with PE [15,39]. IL-1β, activated by Caspase 1, is involved in the induction of inflammatory responses, as well as the generation of oxidative stress [40,41]. Furthermore, IL-1β increases IFN-gamma production by NK cells and is a key cytokine in the differentiation of TH17 cells in human inflammatory conditions [42,43]. In this study, we found that IL-1β was increased in RUPP rats and the inhibition of Caspase 1 with VX-765 significantly reduced placental levels of the proinflammatory cytokine. However, Caspase 1 activity is not solely limited to the production of IL-1β, nor is Caspase-1 limited to the NLRP3 inflammasome. In addition to NLRP3, Caspase 1 is activated by other inflammasomes including NLRP1, IPAF, and NAIPs [34]. Furthermore, Caspase 1 activation also cleaves and activates gasdermin D (GSDMD) [44]. GSDMD activity results in pyroptosis, an inflammatory cell death pathway, which has been shown to be elevated in the placentas of PE women, particularly those suffering from early-onset PE [45]. Investigation of this mechanism was beyond the scope of our study as the RUPP rat is a model of late-onset PE. Nevertheless, we believe we have evidenced that Caspase 1 signaling influences effector immune cell populations via placental inflammatory cytokine secretion. However, the ubiquitous nature of Caspase 1 leaves many avenues to be explored to fully understand its role in PE pathophysiology.

With an estimated logP of 1.6 [46], VX-765 is mostly insoluble in water and is dissolved and administered with DMSO. DMSO is not without effects of its own. DMSO alone can reduce TH17 populations [47]. Administration of DMSO before implantation has also been shown to reduce fetal and placental weight in a mouse model [48]. In our current study, both the treated and control rats received the same amount of DMSO. Therefore, we believe the changes in immune cell populations we see in the RUPP rats to be independent of any DMSO activity since they were not observed in the DMSO-treated NP rats. We also postulate that the late gestational administration of DMSO accounts for the lack of improved fetal and placental weights observed in our study.

The data from the current study implicate Caspase 1 as a key modulator of placental effector TH17 and cNK cell populations and maternal pathophysiological characteristics of a rodent model of placental ischemia. This work serves as a building block for further investigation into potential mechanisms of and therapies for PE.

## 4. Materials and Methods

### 4.1. Animals

Gestation day (GD) 10 or 11 Sprague–Dawley rats were collected from an in-house colony. The animals were randomly assigned to experimental groups and housed in the Center for Comparative Research at the University of Mississippi Medical Center (UMMC). All protocols in this study were in accordance with the National Institutes of Health guidelines for the use and care of animals. Protocols were approved by the UMMC Institutional Animal Care and Use Committee.

### 4.2. Reduced Uterine Perfusion Pressure Surgery

On GD14, a subset of pregnant rats underwent the RUPP surgery under isoflurane anesthesia as previously described [49,50]. After a midline incision was made, the lower abdominal aorta was isolated. A silver clip (0.203 mm) was applied to the aorta superior to the iliac bifurcation to restrict blood flow to the uterine horn. Restrictive clips (0.100 mm) were also applied to branches of the ovarian arteries to reduce compensatory ovarian circulation to the uterus. Another subset of pregnant rats also underwent a Sham surgery of the RUPP rats in which clips were not placed (NP rats). Animals that had complete fetal resorption were excluded from the study, as dictated by our protocol.

### 4.3. Drug Administration

Belnacasan (VX-765) (50 mg/kg/day; Medkoo Biosciences, Inc., Morrisville, NC, USA), a direct inhibitor of Caspase 1 [51], was dissolved in DMSO and administered intraperitoneally immediately after surgery and every 24 h from GD14 to GD18. Control animals received intraperitoneal DMSO injections. Dosing was chosen based on previously published studies [52]. No changes in body weight, behavior, or food intake were observed between vehicle and drug-treated animals.

### 4.4. Uterine Artery Resistance Index Measurement

On GD18, rats underwent Doppler sonography under isoflurane anesthesia and the uterine artery resistance index was measured. A Vevo 770 unit (Visual Sonics, Toronto, ON, Canada), with a 30-Hz transducer (model no. 710B), was used to take Doppler velocimetry measurements of the uterine arteries. One placenta from each uterine horn was imaged to capture waveforms representing the peak systolic velocity (PSV) and end-diastolic flow velocity (EDV). The placenta was visualized midway between the ovary and cervix on both sides. Three waveforms were measured for velocities per frame and averaged. UARI was calculated using the equation UARI = (PSV − EDV)/PSV.

### 4.5. Mean Arterial Pressure Measurement

On GD18, catheters constructed from V3 tubing (BB21785, Scientific Commodities, Lake Havasu City, AZ, USA) were inserted into the carotid arteries and tunneled to the back of the neck under isoflurane anesthesia. On GD19, the rats were placed in individual restrainers. The conscious mean arterial pressure (MAP) was monitored with a pressure transducer (Powerlab, ADInstruments, Colorado Springs, CO, USA) and recorded for 30 min following a 30 min stabilization period.

### 4.6. Sample Collection and Protein Isolation

Rats were anesthetized for blood and tissue collection after MAP measurement. The total litter size and number of live fetuses per litter were recorded. Placentas and fetuses were weighed and recorded for each dam and averaged. Randomly selected placenta tissues (the decidua, basal zone, and labyrinth zone included) were immediately frozen in liquid nitrogen and stored at −80 °C until future analyses. Placenta samples were homogenized in T-PER Tissue Protein Extraction Reagent (78510, Thermo Fisher Scientific, Waltham, MA, USA) containing 1 mM activated vanadate (BP-440, Boston BioProducts, Ashland, MA, USA), protease inhibitor cocktail (P8340, MilliporeSigma, Burlington, MA, USA), and 1× HALT protease and phosphatase inhibitor (78441, Thermo Fisher Scientific, Waltham, MA, USA).

### 4.7. Bio-Plex Singleplex Assay

IL-1β levels were quantified in homogenized placenta samples, using a customized Bio-Plex singleplex immunoassay (Bio-Rad, Hercules, CA, USA), and analyzed in duplicates on a Bio-Rad Bio-Plex 200 system according to the manufacturer’s instructions. Samples were not diluted for the assay. The data were normalized to protein concentration, determined via the Pierce BCA Protein Assay Kit (23225, Thermo Fisher Scientific, Waltham, MA, USA), and expressed as pg/µg. The mean values of technical duplicates are presented.

### 4.8. Western Blot Analysis

Caspase 1 expression was assessed in placental tissue via Western blot. Briefly, placentas were homogenized in cold TPER-buffer and protein extracts, separated by SDS-PAGE, using polyacrylamide gel (4–20%). The proteins were transferred onto nitrocellulose membranes (Bio-Rad) and blocked with Blocking Buffer (LI-COR Biosciences, Lincoln, NE, USA) for 1 h at room temperature. The membranes were incubated overnight at 4 °C with primary antibody directed against Caspase 1 (1:500, Abcam, Waltham, MA, USA), followed by anti-Rabbit IgG HRP secondary antibody (1:1000, R&D, Minneapolis, MN, USA) for 1 h at room temperature, and then scanned using the ChemiDoc MP (Bio-Rad, Hercules, CA, USA). The intensity of specific bands was quantified by densitometry, using Image Lab (Bio-Rad, Hercules, MN, USA), and the expression of Caspase 1 was normalized to total protein, as imaged by Chemidoc MP. All groups were normalized to normal pregnant control values for comparison.

### 4.9. Flow Cytometry

Single-cell suspensions of leukocytes were prepared from the blood and placenta, as previously described [49,53], and blocked with 10% goat and mouse serum before staining for flow cytometry. The antibodies used to stain for NK cells (defined as CD3−ANK61+ cells for the total of NK cells and CD3−ANK61+ ANK44+ cells for the activated NK cells) were as follows: adenomatous polyposis coli protein (APC)-conjugated anti-CD3 (1:10, Miltenyi Biotec San Diego, CA, USA; Cat. No. 130-102-679, RRID:AB_2657097), mouse anti-rat ANK61 (1:50, Abcam, Boston, MA, USA; Cat. No. ab36392, RRID:AB_776652), mouse anti-rat ANK44 (1:100, Abcam, Boston, MA, USA; Cat. No. ab36388, RRID:AB_776651), goat anti-mouse IgG FITC (1:50, Abcam; Cat. No. ab6785, RRID:AB_955241), and rabbit anti-mouse IgG AlexaFluor405 (1:100, Abcam, Boston, MA, USA; Cat. No. ab175651, RRID:AB_2923541). The antibodies used to stain for TH17 cells (defined as CD4+/CD25-/RORγ+) were as follows: FITC-conjugated anti-CD4 antibody (1:10, Miltenyi Biotec San Diego, CA, USA; Cat. No. 130-107-623, RRID:AB_2657928), phycoerythrin (PE)-conjugated anti-CD25 (1:50, BD Biosciences, Franklin Lakes, NJ, USA; Cat. No. 554866, RRID:AB_395564), and peridinin–chlorophyll–protein (PerCP)-conjugated anti-RORγt (1:5, R and D Systems Minneapolis, MN, USA; Cat. No. IC6006C, RRID:AB_10571437). Flow cytometry was carried out on a Miltenyi MACSQuant Analyzer 10 (Miltenyi Biotec, San Diego, CA, USA) and analyzed using FlowLogic software Version 8.6 (Innovai, Sydney, Australia). After dead cell exclusion, using Viobility Fixable dye staining (130-109-814, Miltenyi Biotec, San Diego, CA, USA), lymphocytes were gated in the forward- and side-scatter plots and analyzed with the fluorescence minus one (FMO) controls after doublet exclusion.

### 4.10. Oxidative Stress Measurement

Superoxide, a reactive oxygen species molecule, was measured in homogenized placenta samples, using lucigenin as previously described by our laboratory [49,50]. The homogenized samples were incubated with lucigenin (5 µM) and allowed to equilibrate at 37 °C for 15 min in the dark. Luminescence was then measured for 10 s with a BioTek Plate Reader. An assay blank with only lucigenin was subtracted from the reading. The data were normalized to protein concentration, determined via the Pierce BCA Protein Assay Kit (23225, Thermo Fisher Scientific, Waltham, MA, USA), and expressed as RLU per minute per microgram of protein.

### 4.11. Circulating sFlt and PlGF

sFlt (Abcam, Boston, MA, USA, Cat. No. ab270206) and PlGF (Novus Biologicals, Centennial, CO, Cat. No. MP200) levels were determined in plasma samples of animals from each group via ELISA, in duplicate, following the manufacturer’s protocols. The ratio of sFlt to PlGF for individual animals is presented. The mean values of technical duplicates are presented.

### 4.12. Statistical Analysis

All data are presented as mean ± SD. Statistical analyses were performed using a two-way ANOVA followed by the Tukey’s multiple comparison test in GraphPad Prism 9 (GraphPad Software, San Diego, CA, USA). For the two-way ANOVA analyses, row factors were defined as NP and RUPP and column factors were defined as vehicle treatment and VX-765 treatment. A *p* value < 0.05 was considered statistically significant.

## Data Availability

The data presented in this study are available from the authors upon reasonable request.

## Supplementary Materials

The following supporting information can be downloaded at: https://www.mdpi.com/article/10.3390/ijms25020863/s1.
