# Supplementary material for: Inhibition of Caspase 1 Reduces Blood Pressure, Cytotoxic NK Cells, and Inflammatory T-Helper 17 Cells in Placental Ischemic Rats
Source: Int J Mol Sci. 2024 Jan 10;25(2):863. doi: 10.3390/ijms25020863 (PMC10815407; doi:10.3390/ijms25020863)
Supplement: Supplementary file 1 [file ijms-25-00863-s001.zip › ijms-2671693-supplementary.pdf]

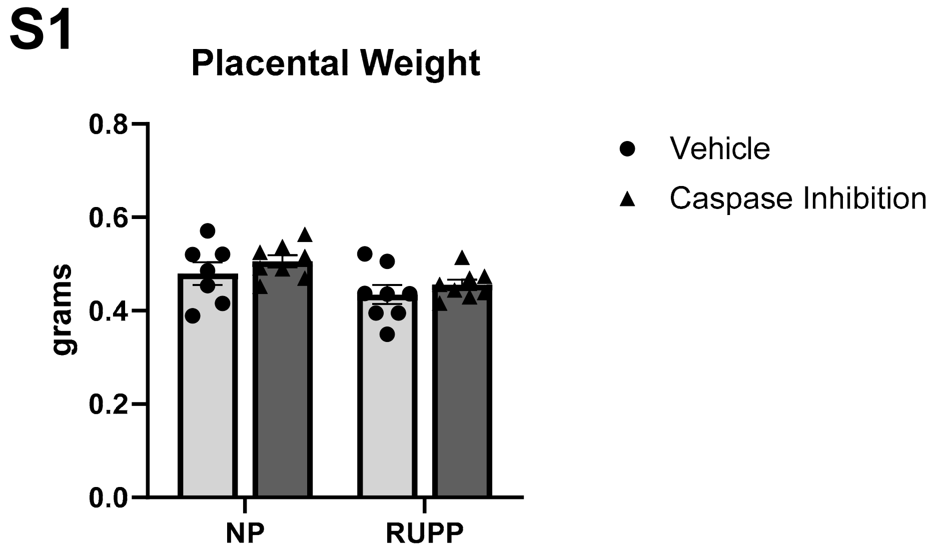

Supplemental Figure S1: Caspase 1 inhibition has no effect on placental weight in NP or RUPP rats. Data presented as Mean ± SEM.

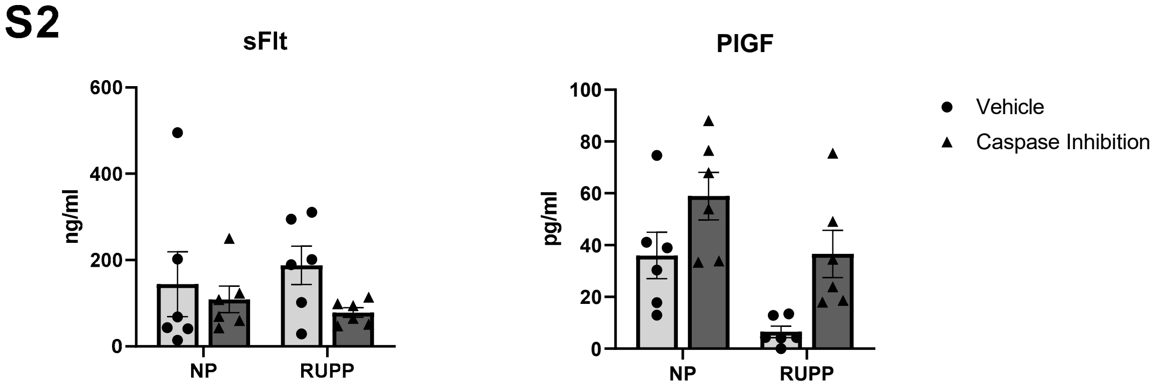

Supplemental Figure S2: No Differences were observed in sFlt or PlGF individually in NP or RUPP rats. Treatment with VX-765 also had no effect. Data are presented as Mean ± SEM.
